# Prevalence, Predictors and Sexual Health Correlates of Heavy Episodic Drinking Among Females of Reproductive Age in Ghana

**DOI:** 10.1002/puh2.70336

**Published:** 2026-08-12

**Authors:** Christian Chukwuma Ndu, Chioma Shallom Ndu, Christa Osei‐Mensah, Adusei Bofa, Georgina Afoakwah

**Affiliations:** ^1^ Department of Nursing and Midwifery Garden City University College Kumasi Ghana; ^2^ Department of Physician Assistantship Garden City University College Kumasi Ghana; ^3^ Department of Business Studies Garden City University College Kumasi Ghana

**Keywords:** alcohol, females, Ghana, heavy episodic drinking, sexual health

## Abstract

**Background:**

Heavy episodic drinking (HED) is a harmful pattern of alcohol use associated with sexual health outcomes. Global prevalence of HED is expected to increase from 20% to 23% by 2030, with females more likely to experience its negative health consequences. This study aimed to determine prevalence, predictors and sexual health correlates of HED among females of reproductive age in Ghana.

**Methods:**

This study employed a cross‐sectional study design that relied on a dataset from the Ghana Demographic and Health Survey, 2022, which consisted of alcohol use history, background characteristics and sexual behaviours and health of 15,014 females. Associations and correlates were identified using chi‐square and binary logistic regression analyses, respectively.

**Results:**

Prevalence of HED among the study population was determined to be 2.1%, with age of 40–44 years (adjusted odds ratio [AOR] = 4.19; 95% confidence interval [CI] = 1.27, 13.87), smoking of cigarettes daily (AOR = 4.46; 95% CI = 1.00, 19.86) and history of terminated pregnancy (AOR = 1.65; 95% CI = 1.24, 2.20) identified as predictors of HED. Females with three extra‐spousal sexual partners (AOR = 6.45; 95% CI = 1.60, 26.10) and females who have had a genital discharge in the last 12 months (AOR = 3.57; 95% CI = 2.19, 5.81) had high odds of engaging in HED.

**Conclusion:**

This study found a low prevalence of HED (2.1%) but identified key high‐risk subgroups. Risky sexual behaviour and adverse sexual health were strongly associated with HED. Public health strategies should consider integrating alcohol screening into sexual and reproductive health services.

## Graphical abstract

Using 2022 Ghana Demographic and Health Survey data from 15,014 females aged 15–49, HED prevalence was 2.1%. Older age, daily smoking and history of terminated pregnancy predicted HED. Multiple extra‐spousal sexual partners and genital discharge are key sexual health correlates. Integrated alcohol–sexual health interventions are recommended.

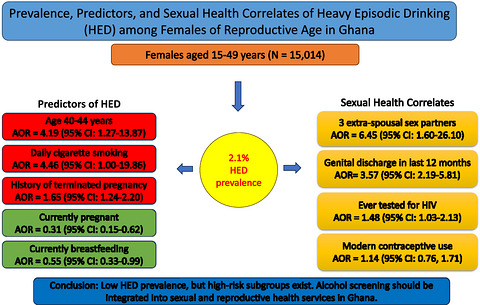

## 1 Introduction

Globally, an estimated 47% of persons aged 15 years and over have been reported to consume alcohol every year, making alcohol one of the most widely used psychoactive substances [1]. Global adult alcohol per capita consumption, defined as the total amount of alcohol consumed per person aged 15 years and over per year, is estimated to be 6.5 L [2], with the average for Africa being 6.4 L [1].

Consumption of alcohol in harmful patterns has been associated with negative health consequences such as noncommunicable diseases (NCDs), mental health disorders, infectious diseases, sexual violence and death [3, 4, 5]. Previous studies conducted in low‐ and high‐income countries have found sex‐ and gender‐based differences in the negative consequences of harmful alcohol use with females and women having increased susceptibility to the detrimental effects of harmful alcohol use as compared to males and men [6, 7, 8, 9].

One of the most hazardous patterns of alcohol use is heavy episodic drinking (HED), defined for women as the consumption of four or more alcoholic drinks on at least one occasion in the past 30 days [1]. In 2017, global prevalence of HED among adults was 20%, and it is expected to increase to 23% by 2030 [2]. Currently, sub‐Saharan Africa (SSA) experiences the highest prevalence of HED among alcohol users globally with more than half of alcohol drinkers (50.2%) in the sub‐region engaging in HED, with prevalence among young females estimated to be 28.2% [1]. This high prevalence in the region adversely impacts the ability of SSA to attain 13 out of the 17 Sustainable Development Goals (SDG) by 2030, particularly SDG 3.5, which targets the strengthening of prevention and treatment of substance abuse, including harmful use of alcohol [10].

Alcohol use among females has also been associated with risky sexual behaviours, including inconsistent condom use and multiple sexual partners, and compromised sexual health outcomes, including sexually transmitted infection (STI), adversely impacting the attainment of SDG 3.7, which aims to ensure universal access to sexual and reproductive health care services, and SDG 5, which targets gender equality and the empowerment of all women and girls [11, 12, 13]. Previous studies have also found risky sexual behaviours, as well as adverse sexual health outcomes, to be associated with harmful alcohol use among females [14, 15, 16].

These findings highlight the complex and bidirectional relationship between alcohol use and sexual health and behaviours [17], which has important implications for public health interventions targeting both alcohol use and sexual health outcomes. Despite this bidirectionality, a substantial body of literature on HED has focused on the prevalence of HED among various populations and HED as a determinant of risky sexual behaviour and adverse sexual health outcomes, with the reverse pathway remaining relatively underexplored [1, 18, 19, 20, 21, 22, 23, 24]. Previous studies in the African sub‐region have identified age, marital status, economic status, residence, tobacco use and level of education as significant correlates of HED but did not explore the reproductive and sexual health correlates of HED [21, 23].

Females in Ghana, a low‐ and middle‐income country (LMIC) in SSA, have a prevalence of alcohol use of 32.4% with current drinkers consuming 5.4 L of alcohol per capita, which has been reported to result in 20.4 alcohol‐attributable deaths per 100,000 females in the country [25]. Few studies have investigated HED among females in Ghana and the associations of sexual health outcomes with HED. This gap is concerning given that prevalence of human immunodeficiency virus (HIV) and STI among females in Ghana has been estimated to be 2.5% and 12.5%–19.5%, respectively [26, 27, 28, 29], highlighting the need to understand how this could potentially influence HED among females in Ghana. The dearth in literature hinders the formulation and implementation of effective policy measures aimed at reducing alcohol exposure and improving sexual health among females in Ghana [1].

This study, therefore, sought to determine the prevalence, predictors and sexual health correlates of HED among females of reproductive age in Ghana. The findings from this study can potentially inform the development of integrated public health interventions that simultaneously address alcohol use and adverse sexual health among females of reproductive age in Ghana and similar LMIC settings.

## 2 Methods

### 2.1 Study Design

This study employed a quantitative cross‐sectional design employing a dataset from the 2022 Ghana Demographic and Health Survey (GDHS) [30]. The data used for analysis were obtained from female participants, collected by the Ghana Statistical Service from 17 October 2022 to 14 January 2023.

### 2.2 Study Area

Ghana is located in West Africa, with a total land area of 238,537 km^2^. As of 2021, the country had a population of 30,832,019 with males accounting for 15,200,440 (49.3%) and females, 15,631,579 (50.7%) [31].

### 2.3 Study Population

The study population consisted of Ghanaian females aged 15–49 years who were surveyed in the 2022 GDHS. The data were collected using structured questionnaires that were administered through face‐to‐face interviews.

### 2.4 Inclusion Criteria

All Ghanaian females aged 15–49 years.

### 2.5 Exclusion Criteria

Ghanaian females younger than 15 years and older than 49 years were excluded from the study. Females who did not consent or were critically sick at the time of the data collection were omitted from the study.

### 2.6 Sampling Technique and Sample Size

The GHIR8AFL dataset from the 2022 GDHS was utilised [30]. These secondary data, obtained from a large and nationally representative sample determined through stratified multistage cluster sampling, required no separate a priori sample size calculation. This dataset used was obtained from interviewing 15,014 females aged 15–49 years and had 5581 variables. In order to achieve this study's objectives, 21 variables were selected: age in 5‐year groups; type of place of residence; highest educational level; self‐reported health status; wealth index; currently pregnant; ever had a terminated pregnancy; total children ever born; current use of contraceptive by method type; currently breastfeeding; frequency smokes cigarettes; number of alcoholic drinks per day; current marital status; number of sex partners, excluding spouse, in the last 12 months; ever been tested for HIV; had any STI in the last 12 months; had genital sore/ulcer in the last 12 months; had genital discharge in the last 12 months; women's individual sample weight (6 decimals); primary sampling unit and sample strata for sampling errors. These variables did not have any missing values; hence, this study analysed data from all the 15,014 respondents interviewed, implying a retention of 100% of the initial sample for robust statistical analysis.

### 2.7 Dependent Variables

The dependent variable of this study was HED, defined as the consumption of four or more alcoholic drinks on at least one typical drinking day in the past 30 days (No = 0, Yes = 1) [24, 32]. HED status was derived from the ‘number of alcoholic drinks consumed per day in the past 30 days’ variable in the 2022 GDHS dataset. Respondents were asked, ‘On the days that you drank alcohol during the last one month, how many drinks did you usually have per day?’ This question allowed for the estimation of usual quantity of drinks consumed per drinking day but did not permit the calculation of weekly alcohol intake.

The 2022 GDHS questionnaire did not provide standardised definitions or examples of a standard drink, meaning that the term ‘drink’ was not standardised. Responses to the alcohol consumption question therefore reflect self‐reported behaviour as understood by the respondents.

### 2.8 Independent Variables

The independent variables of this study were selected a priori on the basis of evidence from previous literature demonstrating their theoretical and empirical relevance to HED among females of reproductive age. Most independent variables, except parity, were adopted as reported in the 2022 GDHS dataset and are described below:

Predictor variables:

- Age of females (15–49 years): reported as 15–19, 20–24, 25–29, 30–34, 35–39, 40–44 and 45–49.
- Type of place of residence: reported as urban and rural as captured in the 2022 GDHS.
- Highest educational level: reported as No education, primary, secondary and higher.
- Wealth index: reported as Poorest, Poorer, Middle, Richer and Richest.
- Current marital status: reported as Never in union, Married, Living with partner, Widowed, Divorced and No longer living together/separated.
- Currently pregnant: reported as No or unsure and Yes.
- Ever had a terminated pregnancy: reported as No and Yes.
- Currently breastfeeding: reported as No and Yes.
- Parity: recoded from the ‘total children ever born’ variable in the 2022 GDHS. Females were classified as nulliparous (females who have not borne any children), uniparous (females who have borne only one child) and multiparous (females who have borne more than one child).
- Frequency smokes cigarettes: categorised as does not smoke, some days or every day.

Sexual health and behaviours correlates were measured as follows:

- Self‐reported health status: reported as Very Good, Good, Moderate, Bad and Very bad.
- Had any STI in the last 12 months: reported as No, Yes and Do not know.
- Had genital sore/ulcer in the last 12 months: reported as No, Yes and Do not know.
- Had genital discharge in the last 12 months: reported as No, Yes and Do not know.
- Ever been tested for HIV: reported as No and Yes.
- Number of sex partners, excluding spouse, in the last 12 months: recorded as none, one, two or three.
- Current contraceptive use by type: categorised as no method, folkloric method, traditional method and modern method.

### 2.9 Data Analysis

Results were presented in tables using descriptive statistics (frequencies and percentages). To explore associations between the dependent and independent variables (bivariate analysis), chi‐square analysis was used, and independent variables were subsequently subjected to binary logistic regression analysis, which was used to determine the predictors of the dependent variable. Binary logistic regression was presented as crude odds ratio (COR) and adjusted odds ratio (AOR) in order to control for any confounding variables, with corresponding 95% confidence interval (CI). All independent variables selected a priori were included in the multivariable logistic regression model to adjust for potential confounders. Separate models were run for sociodemographic factors and sexual health and behaviour factors. Statistical significance was set at *p* < 0.05. Model fit and multicollinearity were assessed using Hosmer–Lemeshow test and variance inflation factors, respectively. All statistical analyses conducted incorporated GDHS sample weights, primary sampling units and stratification of variables using the complex sample module in SPSS version 27.

### 2.10 Ethical Considerations

Ethical approval was not necessary for this study because it involved secondary analysis of a dataset with no revelation of the identity of the respondents or their household. Approval of the protocol for the use of the 2022 GDHS report was obtained from Ghana Statistical Service. The dataset is publicly available and also fully anonymised prior to its release; hence, no personally identifiable information was accessible to the authors of this study.

## 3 Results

### 3.1 Characteristics of Respondents

Out of the 15,014 respondents, 57.0% of them reside in urban communities, do not smoke cigarettes (99.1%), have never had a terminated pregnancy (74.7%) and were neither currently breastfeeding (80.5%) nor pregnant (93.2%) (Table 1).

**TABLE 1:** Characteristics of study participants (*N* = 15,014).

Characteristics	Weighted frequency (*N*)	Weighted percentage
**Age**		
15–19	2682	17.9
20–24	2695	17.9
25–29	2339	15.6
30–34	2252	15.0
35–39	2059	13.7
40–44	1675	11.2
45–49	1312	8.7
**Type of place of residence**		
Urban	8557	57.0
Rural	6457	43.0
**Highest educational level**		
No education	2411	16.1
Primary	2070	13.8
Secondary	8999	59.9
Higher	1534	10.2
**Current marital status**		
Never in union	5268	35.1
Married	6007	40.0
Living with partner	2197	14.6
Widowed	366	2.4
Divorced	389	2.6
No longer living together/separated	787	5.2
**Wealth index**		
Poorest	2447	16.3
Poorer	2712	18.1
Middle	3121	20.8
Richer	3379	22.5
Richest	3355	22.3
**Currently pregnant**		
No or unsure	13,989	93.2
Yes	1025	6.8
**Ever had a terminated pregnancy**		
No	11,217	74.7
Yes	3797	25.3
**Currently breastfeeding**		
No	12,089	80.5
Yes	2925	19.5
**Frequency smokes cigarettes**		
Does not smoke	14,882	99.1
Every day	16	0.1
Some days	116	0.8
**Parity**		
Nulliparous	4854	32.3
Uniparous	2421	16.1
Multiparous	7739	51.5

### 3.2 Prevalence and Associations of HED

As shown in Table 2, the overall prevalence of HED among females of reproductive age in Ghana was 2.1% (1.7, 2.5). Prevalence of HED were highest among females who smoke cigarettes every day (6.0%), females aged 40–44 years (4.7%), and females who have separated from their partners (4.6%). Pregnant (0.7%), nulliparous (0.9%) and females with higher education (1.0%) had some of the lowest prevalence of HED. Age, marital status, parity, pregnancy and breastfeeding, and history of terminated pregnancy were characteristics significantly associated with HED.

**TABLE 2:** Bivariate analysis of factors associated with heavy episodic drinking among study participants (*N* = 15,014).

Characteristics	No		Yes		
	*n*	% (95% CI)	*n*	% (95% CI)	*p* value
All	14,704	97.9 (97.5, 98.3)	310	2.1 (1.7, 2.5)	
**Age**					<0.001
15–19	2661	99.2 (98.7, 99.5)	21	0.8 (0.5, 1.3)	
20–24	2667	99.0 (98.3, 99.4)	28	1.0 (0.6, 1.7)	
25–29	2294	98.1 (97.2, 98.7)	45	1.9 (1.3, 2.8)	
30–34	2202	97.8 (96.3, 98.7)	50	2.2 (1.3, 3.7)	
35–39	2012	97.7 (96.7, 98.5)	47	2.3 (1.5, 3.3)	
40–44	1596	95.3 (93.4, 96.7)	79	4.7 (3.3, 6.6)	
45–49	1272	96.9 (95.3, 98.0)	40	3.1 (2.0, 4.7)	
**Type of place of residence**					0.693
Urban	8375	97.9 (97.1, 98.4)	182	2.1 (1.6, 2.9)	
Rural	6329	98.0 (97.5, 98.4)	128	2.0 (1.6, 2.5)	
**Highest educational level**					0.108
No education	2355	97.7 (96.6, 98.4)	56	2.3 (1.6, 3.4)	
Primary	2020	97.6 (96.7, 98.2)	50	2.4 (1.8, 3.3)	
Secondary	8811	97.9 (97.3, 98.4)	188	2.1 (1.6, 2.7)	
Higher	1518	99.0 (98.1, 99.5)	16	1.0 (0.5, 1.9)	
**Current marital status**					0.002
Never in union	5191	98.5 (98.0, 99.0)	77	1.5 (1.0, 2.0)	
Married	5892	98.1 (97.4, 98.6)	115	1.9 (1.4, 2.6)	
Living with partner	2139	97.4 (95.8, 98.3)	58	2.6 (1.7, 4.2)	
Widowed	354	96.6 (91.9, 98.7)	12	3.4 (1.3, 8.1)	
Divorced	378	97.1 (94.1, 98.6)	11	2.9 (1.4, 5.9)	
No longer living together/separated	750	95.4 (92.8, 97.0)	37	4.6 (3.0, 7.2)	
**Wealth index**					0.949
Poorest	2402	98.2 (97.4, 98.7)	45	1.8 (1.3, 2.6)	
Poorer	2660	98.1 (97.3, 98.6)	52	1.9 (1.4, 2.7)	
Middle	3053	97.8 (96.9, 98.5)	68	2.2 (1.5, 3.1)	
Richer	3307	97.9 (96.7, 98.7)	72	2.1 (1.3, 3.3)	
Richest	3282	97.8 (96.6, 98.6)	73	2.2 (1.4, 3.4)	
**Currently pregnant**					<0.001
No or unsure	13,686	97.8 (97.3, 98.2)	303	2.2 (1.8, 2.7)	
Yes	1018	99.3 (98.8, 99.7)	7	0.7 (0.3, 1.2)	
**Ever had a terminated pregnancy**					<0.001
No	11,042	98.4 (98.0, 98.8)	175	1.6 (1.2, 2.0)	
Yes	3662	96.5 (95.5, 97.2)	135	3.5 (2.8, 4.5)	
**Currently breastfeeding**					0.027
No	11,819	97.8 (97.2, 98.2)	270	2.2 (1.8, 2.8)	
Yes	2885	98.7 (97.9, 99.1)	40	1.3 (0.9, 2.1)	
**Frequency smokes cigarettes**					0.050
Does not smoke	14,573	97.9 (97.5, 98.3)	309	2.1 (1.7, 2.5)	
Every day	15	94.0 (75.5, 98.8)	1	6.0 (1.2, 24.5)	
Some days	116	100 (97.9, 100.0)			
**Parity**					<0.001
Nulliparous	4810	99.1 (98.6, 99.4)	44	0.9 (0.6, 1.4)	
Uniparous	2377	98.2 (97.2, 98.8)	44	1.8 (0.4, 2.8)	
Multiparous	7517	97.1 (96.4, 97.7)	222	2.9 (2.3, 3.6)	

*Note:* *n*—weighted frequency of variable; %—weighted percentage of frequency.

Abbreviation: CI, confidence interval.

### 3.3 Predictors of HED

It was observed that the odds of engaging in HED was highest among females who smoke cigarettes every day (AOR = 4.46; 95% CI = 1.00, 19.86), females aged 40–44 years (AOR = 4.19; 95% CI = 1.27, 13.87) and multiparous females (AOR = 3.27; 95% CI = 1.30, 8.23). As compared to females who have never been in a union, females of all other marital statuses were less likely to engage in HED. Females currently breastfeeding (AOR = 0.55; 95% CI = 0.33, 0.90) and pregnant (AOR = 0.31; 95% CI = 0.15, 0.62) were less likely to engage in HED. The odds of HED were also found to be high among females who have ever had a terminated pregnancy (AOR = 1.65; 95% CI = 1.24, 2.20) and females of the highest economic status (AOR = 1.28; 95% CI = 0.68, 2.42) (Table 3).

**TABLE 3:** Multivariable logistic regression analysis of factors associated with heavy episodic drinking among study participants (*N* = 15,014).

Characteristics	Crude			Adjusted		
	*p* value	COR	95% CI	*p* value	AOR^a^	95% CI
**Age**	<0.001			0.130		
15–19		Reference			Reference	
20–24		1.33	0.67, 2.63		1.28	0.60, 2.72
25–29		2.50	1.35, 4.63		2.30	0.90, 5.87
30–34		2.86	1.33, 6.12		2.47	0.68, 8.93
35–39		2.93	1.54, 5.59		2.22	0.73, 6.80
40–44		6.24	3.45, 11.31		4.19	1.27, 13.87
45–49		3.99	2.12, 7.51		2.55	0.85, 7.64
**Type of place of residence**	0.693			0.973		
Urban		Reference			Reference	
Rural		0.92	0.62, 1.37		1.01	0.66, 1.53
**Highest educational level**	0.117			0.179		
No education		Reference			Reference	
Primary		1.05	0.64, 1.73		1.03	0.61, 1.72
Secondary		0.90	0.59, 1.37		1.02	0.61, 1.70
Higher		0.43	0.20, 0.94		0.46	0.18, 1.15
**Current marital status**	<0.001			0.008		
Never in union		Reference			Reference	
Married		1.33	0.89, 1.98		0.34	0.19, 0.62
Living with partner		1.83	1.05, 3.22		0.58	0.27, 1.22
Widowed		2.35	0.91, 6.06		0.41	0.15, 1.16
Divorced		2.04	0.97, 4.26		0.36	0.14, 0.91
No longer living together/separated		3.30	2.01, 5.41		0.74	0.38, 1.43
**Wealth index**	0.933			0.871		
Poorest		Reference			Reference	
Poorer		1.06	0.65, 1.71		0.90	0.56, 1.44
Middle		1.20	0.76, 1.89		0.98	0.61, 1.56
Richer		1.17	0.64, 2.13		0.98	0.57, 1.68
Richest		1.20	0.67, 2.15		1.28	0.68, 2.42
**Currently pregnant**	<0.001			0.001		
No or unsure		Reference			Reference	
Yes		0.30	0.15, 0.58		0.31	0.15, 0.62
**Ever had a terminated pregnancy**	<0.001			<0.001		
No		Reference			Reference	
Yes		2.32	1.78, 3.02		1.65	1.24, 2.20
**Currently breastfeeding**	0.029			0.018		
No		Reference			Reference	
Yes		0.59	0.37, 0.95		0.55	0.33, 0.90
**Frequency smokes cigarettes**	0.064			0.028		
Does not smoke		Reference			Reference	
Every day		3.00	0.58, 15.52		4.46	1.00, 19.86
Some days		0.14	0.02, 1.03		0.16	0.02, 1.20
**Parity**	<0.001			0.042		
Nulliparous		Reference			Reference	
Uniparous		1.96	1.14, 3.39		2.17	1.02, 4.64
Multiparous		3.18	2.09, 4.83		3.27	1.30, 8.23

Abbreviations: AOR, adjusted odds ratio; CI, confidence interval; COR, crude odds ratio.

^a^ The final model (adjusted model) included age, type of residence, highest educational level, current marital status, wealth index, currently pregnant, ever had a terminated pregnancy, currently breastfeeding, frequency smokes cigarettes and parity.

### 3.4 Associations Between HED, Sexual Behaviour and Sexual Health

As shown in Table 4, prevalence of HED was found to generally rise with worsening self‐reported health status, with females with very bad health status having a prevalence of 5.0%. It was also observed that HED was more prevalent among females who used modern contraceptive methods (2.8%), as compared to females who used traditional methods (2.2%) or no method (1.8%) of contraceptive. It was found that HED became more prevalent with an increasing number of extra‐spousal sexual partners, peaking among females with three (10.9%) extra‐spousal sexual partners. As compared to females with no extra‐spousal sexual partners, the odds of engaging in HED were found to rise with increasing number of extra‐spousal sexual partners, with females with three other sexual partners besides their spousal (AOR = 6.45; 95% CI = 1.60, 26.10) being 6.5 times more likely to engage in HED. Females who have ever been tested for HIV (2.4%; AOR = 1.48; 95% CI = 1.03, 2.13) had a high prevalence of and were 1.5 times more likely to engage in HED. Females who have had genital discharge in the last 12 months (AOR = 3.57; 95% CI = 2.19, 5.81) had significantly higher odds of engaging in HED (Table 5).

**TABLE 4:** Bivariate analysis of sexual behaviours and sexual health outcomes associated with heavy episodic drinking among study participants (*N* = 15,014).

Sexual health outcomes and behaviours	No		Yes		
	*n*	% (95% CI)	*n*	% (95% CI)	*p* value
**Self‐reported health status**					0.108
Very good	4569	97.6 (96.6, 98.3)	112	2.4 (1.7, 3.4)	
Good	6752	98.4 (97.8, 98.8)	110	1.6 (1.2, 2.2)	
Moderate	2889	97.7 (96.6, 98.4)	68	2.3 (1.6, 3.4)	
Bad	434	96.2 (93.4, 97.8)	17	3.8 (2.2, 6.6)	
Very bad	60	95.0 (72.0, 99.3)	3	5.0 (0.7, 28.0)	
**Current contraceptive use by method type**					0.110
No method	10,099	98.2 (97.6, 98.6)	190	1.8 (1.4, 2.4)	
Folkloric method	160	100 (100, 100)			
Traditional method	1023	97.8 (96.1, 98.8)	23	2.2 (1.2, 3.9)	
Modern method	3422	97.2 (96.3, 98.0)	97	2.8 (2.0, 3.7)	
**Number of sex partners, excluding spouse, in the last 12 months**					<0.001
None	11,362	98.3 (97.9, 98.7)	194	1.7 (1.3, 2.1)	
One	3104	96.9 (95.6, 97.8)	100	3.1 (2.2, 4.4)	
Two	208	94.4 (88.2, 97.5)	12	5.6 (2.5, 11.8)	
Three	30	89.1 (68.5, 96.9)	4	10.9 (3.1, 31.5)	
**Ever been tested for HIV**					0.023
No	6299	98.4 (97.8, 98.8)	104	1.6 (1.2, 2.2)	
Yes	8405	97.6 (97.0, 98.1)	206	2.4 (1.9, 3.0)	
**Had any STI in the last 12 months**					0.861
No	13,895	98.0 (97.5, 98.3)	290	2.0 (1.7, 2.5)	
Yes	803	97.6 (95.2, 98.8)	20	2.4 (1.2, 4.8)	
Do not know	6	100 (100, 100)			
**Had genital sore/ulcer in the last 12 months**					0.536
No	13,553	98.0 (97.5, 98.4)	280	2.0 (1.6, 2.5)	
Yes	1147	97.5 (95.7, 98.5)	30	2.5 (1.5, 4.3)	
Do not know	4	100 (100, 100)			
**Had genital discharge in the last 12 months**					<0.001
No	12,012	98.6 (98.2, 98.9)	175	1.4 (1.1, 1.8)	
Yes	2693	95.3 (93.4, 96.6)	135	4.7 (3.4, 6.6)	

*Note:* *n*—weighted frequency of variable; %—weighted percentage of frequency; *N*—weighted population of variable.

Abbreviations: CI, confidence interval; HIV, human immunodeficiency virus; STI, sexually transmitted infection.

**TABLE 5:** Multivariable logistic regression analysis of sexual behaviours and sexual health outcomes associated with heavy episodic drinking among study participants (*N* = 15,014).

Sexual health outcomes and behaviours	Crude			Adjusted		
	*p* value	COR	95% CI	*p* value	AOR^a^	95% CI
**Self‐reported health status**	0.032			0.053		
Very good		Reference			Reference	
Good		0.67	0.41, 1.10		0.66	0.40, 1.07
Moderate		0.97	0.58, 1.63		0.90	0.54, 1.50
Bad		1.64	0.83, 3.24		1.58	0.77, 3.24
Very bad		2.18	0.29, 16.55		2.19	0.27, 17.66
**Current contraceptive use by method type**	<0.001			<0.001		
No method		Reference			Reference	
Traditional method		1.18	0.64, 2.18		0.81	0.42, 1.54
Modern method		1.51	1.07, 2.12		1.14	0.76, 1.71
**Number of sex partners, excluding spouse, in the last 12 months**	<0.001			0.004		
None		Reference			Reference	
One		1.89	1.25, 2.85		1.63	1.04, 2.53
Two		3.45	1.50, 7.93		2.98	1.23, 7.25
Three		7.15	1.88, 27.13		6.45	1.60, 26.10
**Ever been tested for HIV**	0.024			0.032		
No		Reference			Reference	
Yes		1.47	1.05, 2.06		1.48	1.03, 2.13
**Had any STI in the last 12 months**	0.891			0.979		
No		Reference			Reference	
Yes		1.19	0.87, 1.77		0.65	0.41, 0.90
Do not know		0.00	0.00		0.00	0.00
**Had genital sore/ulcer in the last 12 months**	0.997			0.945		
No		Reference			Reference	
Yes		1.26	1.00, 1.81		0.57	0.38, 0.72
Do not know		0.00	0.00		0.00	0.00
**Had genital discharge in the last 12 months**	<0.001			<0.001		
No		Reference			Reference	
Yes		3.41	2.29, 5.09		3.57	2.19, 5.81

Abbreviations: AOR, adjusted odds ratio; CI, confidence interval; COR, crude odds ratio.

^a^ The final model (adjusted model) included self‐reported health status; current contraceptive use by method type; number of sex partners, excluding spouse, in the last 12 months; ever been tested for HIV; had any STI in the last 12 months; had genital sore/ulcer in the last 12 months and had genital discharge in the last 12 months.

## 4 Discussion

This study sought to determine the prevalence and correlates of HED among females of reproductive age in Ghana and associations of this drinking pattern with sexual behaviours and sexual health.

### 4.1 Prevalence of HED

This study found the prevalence of HED to be 2.1%, which is less than the prevalence reported in Ecuador (12.0%), Ethiopia (11.5%) and the global average of 20% [2, 22, 23]. This relatively low prevalence could potentially be due to the effectiveness of the national alcohol policy, which was operationalised in 2016 and aimed to reduce harmful alcohol use among females of reproductive age [33]. Although prevalence of HED was low, alcohol use by females is likely to significantly elevate their risk of negative health consequences such as NCDs and intimate partner violence, as well as negative foetal and neonatal health outcomes [6, 7, 8, 34].

### 4.2 Predictors of HED

This study found different age groups to have differing odds of HED. This suggests the existence of age‐specific differences in factors that increase the likelihood of HED. Previous studies that investigated age as a determinant of alcohol use have found that younger and middle‐aged adults faced varying factors that elevated their risk of alcohol use [35, 36, 37]. Predictors of harmful alcohol use among younger adults were found to be anxiety and depression, social interactions and loss of inhibitory control and impulsive decision‐making, whereas socioeconomic status, life stress, marital status, childhood adversity and mood were predictors promoting harmful alcohol use among middle‐aged adults [35, 36, 37].

Although not statistically significant, the odds of HED were observed in this study to reduce with increasing educational status. Educated females may be better able to resist HED, as they likely are informed about its negative health, social and economic effects. This finding is consistent with that found in a previous study on alcohol use among women in Ghana [26].

Marital status was identified in this study to be associated with HED, where married and divorced females were found to be significantly unlikely to engage in HED. This finding is contrary to those reported by previous studies on HED among adults in Kenya [21] and alcohol use among females in Ghana, which used data collected in 2008 before the institution of the national alcohol policy [26]. These differences may be attributed to the effective implementation of the national alcohol policy and education of females of the harmful effects of alcohol use.

Although breastfeeding and pregnant females were significantly less likely to engage in HED, this study found prevalence of this drinking pattern to be 1.3% and 0.7%, respectively. This intake of harmful amounts of alcohol exposes neonates and foetuses to morbidities and mortalities as alcohol can enter breastmilk and cross the placental barrier [34, 38, 39]. Harmful exposure of neonates and foetuses to alcohol has been found to be a risk factor that elevates the odds of neonatal low birth weight, neonatal respiratory distress, neonatal difficulty breastfeeding and foetal alcohol syndrome [38, 39]. The risk of alcohol use among pregnant and breastfeeding females has been reported to be associated with younger maternal age, fewer antenatal visits, being primiparous and having a history of mental illness and substance use [38]. These findings highlight the urgent need for strategies that reduce alcohol use among pregnant and breastfeeding females.

This study also found the odds of HED to be high among females who have ever had a terminated pregnancy. This finding is consistent with that of previous studies, which investigated links between alcohol use and mental health in pregnancy and found that the termination of pregnancy elevates the risk of mental health disorders like anxiety, depression, suicidal ideations and substance abuse [8, 40, 41]. It was also found in this study that females who smoke cigarettes every day had high odds of engaging in HED. Although statistically significant, the wide CI of this variable reflects the small number of daily smokers in the sample and warrants cautious interpretation. This finding is consistent with previous studies, which found tobacco use to be a reliable predictor of harmful use of alcohol as each substance potentiates the consumption of the other [12, 23, 41].

As compared to nulliparous females, uniparous and multiparous females had higher odds of HED, and this is possibly associated with the use of alcohol as a coping mechanism by uniparous and multiparous females in dealing with intimate partner violence that usually result from economic stress, reduced autonomy and marital dissatisfaction [42, 43].

### 4.3 Sexual Health Correlates of HED

This study found that sexual behaviours and sexual health had mixed effects on the odds of HED. Having extra‐spousal sexual partners, a high‐risk sexual behaviour that increases the chances of STIs [44], was the only risky sexual behaviour explored in this study that was linked to high odds HED. Having ever been tested for HIV and using modern methods of contraception, which are not risky sexual behaviours, were also associated with high odds of HED. These findings were contrary to those reported in previous studies on alcohol use and sexual behaviours [14, 45, 46]. This could potentially be due to females who engage in HED having a heightened perception of HIV and unintended pregnancy risks, leading them to seek HIV testing and adopt modern contraception as a protective measure. In Ghana, where HIV and STI burden remain substantial, these findings underscore the need to integrate alcohol screening into sexual and reproductive health services.

Although no cause‐and‐effect relationship can be established by this study, females who reported bad and very bad health statuses, or who had genital discharge in the last 12 months, had higher odds of HED. These indicators suggest that poor sexual health outcomes among females of reproductive age were associated with HED, and this finding is consistent with existing literature [12, 47, 48]. Self‐reported health, an indicator of general wellbeing that includes reproductive and sexual health, is a known predictor of morbidity and mortality [49]. The association between poor self‐reported health and HED observed in this study aligns with findings of a previous research, which investigated this association among Canadian and Chinese adults [49]. Although not formally tested, the co‐occurrence of adverse sexual health outcomes and HED may reflect syndemic interaction, where both conditions potentiate each other, suggesting possible bidirectional pathways between both conditions [50].

### 4.4 Limitations

This study is limited by its cross‐sectional design, which limits the ability to make causal inferences, and by reliance on self‐reported data, which may introduce reporting bias. This study is also limited by the collapsing of some variable categories, which might have affected their significance or otherwise, and by the possibility that data collected in 2022–2023 may not reflect current patterns. The use of nationally representative, weighted dataset from 2022 GDHS enhances the generalisability of findings to women aged 15–49 years in Ghana during the survey.

### 4.5 Implications and Recommendations

The findings of this study emphasise the need for integrated interventions that simultaneously mitigate HED, risky sexual behaviour and poor sexual health. By identifying females aged 40–44 years, multiparous females, daily smokers and those with history of terminated pregnancy as high‐risk subgroups, this study provides clear targets for preventive measures. Although pregnancy and breastfeeding females had lower odds of HED, any pattern of alcohol use by these groups warrant continued attention.

This study recommends that the national alcohol policy be strengthened to restrict alcohol sale to pregnant and breastfeeding women, limit advertising and raise the legal purchasing age. Alcohol screening should be incorporated into sexual and reproductive health services, including antenatal and postnatal care, family planning clinics and HIV testing. Routine mental health assessment in antenatal and postnatal care would be useful in identifying women at risk of harmful alcohol use. Public health campaigns leveraging social media should be used to deliver integrated messages on alcohol reduction and safer sexual practices. Future longitudinal research is, however, needed to explore causal pathways and formally test syndemic interactions between HED, sexual behaviours and sexual health outcomes.

## 5 Conclusion

This study found a low prevalence of HED (2.1%) among females of reproductive age in Ghana but identified key high‐risk subgroups. Risky sexual behaviour and adverse sexual health were strongly associated with HED. Public health strategies should consider integrating alcohol screening into sexual and reproductive health services. Future research should employ longitudinal designs to explore causal pathways and formally test synergistic interactions between sexual health outcomes and HED.

## Author Contributions

Christian Chukwuma Ndu contributed to the conceptualisation of the study, analysis and interpretation of data, and drafted and substantially revised article. Chioma Shallom Ndu contributed to the conceptualisation of the study, interpretation of data, and drafted and substantially revised article. Christa Osei‐Mensah drafted and revised the article. Adusei Bofa analysed, drafted and substantially revised article. Georgina Afoakwah contributed to the conceptualisation of the study and drafted and substantially revised article.

## Funding

The authors have nothing to report.

## Ethics Statement

The authors have nothing to report.

## Consent

The authors have nothing to report.

## Conflicts of Interest

The authors declare no conflicts of interest.

## Data Availability

The data that support the findings of this study are openly available in Ghana Statistical Service Microdata Catalog at https://microdata.statsghana.gov.gh/index.php/home, reference number GHA_2022_DHS_v01_M.
